# Changes in DNA methylation profile in liver tissue during progression of HCV-induced fibrosis to hepatocellular carcinoma

**DOI:** 10.18699/VJGB-23-10

**Published:** 2023-03

**Authors:** I.A. Goncharova, A.A. Zarubin, N.P. Babushkina, I.A. Koroleva, M.S. Nazarenko

**Affiliations:** Research Institute of Medical Genetics, Tomsk National Research Medical Center of the Russian Academy of Sciences, Tomsk, Russia; Research Institute of Medical Genetics, Tomsk National Research Medical Center of the Russian Academy of Sciences, Tomsk, Russia; Research Institute of Medical Genetics, Tomsk National Research Medical Center of the Russian Academy of Sciences, Tomsk, Russia; Research Institute of Medical Genetics, Tomsk National Research Medical Center of the Russian Academy of Sciences, Tomsk, Russia; Research Institute of Medical Genetics, Tomsk National Research Medical Center of the Russian Academy of Sciences, Tomsk, Russia

**Keywords:** DNA methylation, chronic hepatitis C, HCV, liver fibrosis, liver cirrhosis, hepatocellular carcinoma, метилирование ДНК, ХВГС, фиброз печени, цирроз печени, гепатоцеллюлярная карцинома

## Abstract

In this study we compared methylation levels of 27,578 CpG sites between paired samples of the tumor and surrounding liver tissues with various degrees of damage (fibrosis, cirrhosis) in HCV-induced hepatocellular carcinoma (HCC) patients, as well as between tumor and normal tissue in non-viral HCC patients, using GSE73003 and GSE37988 data from GEODataSets (https://www.ncbi.nlm.nih.gov/). A significantly lower number of differentially methylated sites (DMS) were found between HCC of non-viral etiology and normal liver tissue, as well as between HCC and fibrosis (32 and 40), than between HCC and cirrhosis (2450 and 2304, respectively, according to GSE73003 and GSE37988 datasets). As the pathological changes in the tissue surrounding the tumor progress, the ratio of hyper-/hypomethylated DMSs in the tumor decreases. Thus, in tumor tissues compared with normal/fibrosis/cirrhosis of the liver, 75/62.5/47.7 % (GSE73003) and 16 % (GSE37988) of CpG sites are hypermethylated, respectively. Persistent hypermethylation of the ZNF154 and ZNF540 genes, as well as CCL20 hypomethylation, were registered in tumor tissue in relation to both liver fibrosis and liver cirrhosis. Protein products of the EDG4, CCL20, GPR109A, and GRM8 genes, whose CpG sites are characterized by changes in DNA methylation level in tumor tissue in the setting of cirrhosis and fibrosis, belong to “Signaling by G-protein-coupled receptors (GPCRs)” category. However, changes in the methylation level of the “driver” genes for oncopathology (АРС, CDKN2B, GSTP1, ELF4, TERT, WT1) are registered in tumor tissue in the setting of liver cirrhosis but not fibrosis. Among the genes hypermethylated in tumor tissue in the setting of liver cirrhosis, the most represented biological pathways are developmental processes, cell-cell signaling, transcription regulation, Wnt-protein binding. Genes hypomethylated in liver tumor tissue in the setting of liver cirrhosis are related to olfactory signal transduction, neuroactive ligand-receptor interaction, keratinization, immune response, inhibition of serine proteases, and zinc metabolism. The genes hypermethylated in the tumor are located at the 7p15.2 locus in the HOXA cluster region, and the hypomethylated CpG sites occupy extended regions of the genome in the gene clusters of olfactory receptors (11p15.4), keratin and keratin-associated proteins (12q13.13, 17q21.2, and 21q22.11), epidermal differentiation complex (1q21.3), and immune system function loci 9p21.3 (IFNA, IFNB1, IFNW1 cluster) and 19q13.41–19q13.42 (KLK, SIGLEC, LILR, KIR clusters). Among the genes of fibrogenesis or DNA repair, cg14143055 (ADAMDEC1) is located in the binding region of the HOX gene family transcription factors (TFs), while cg05921699 (CD79A), cg06196379 (TREM1) and cg10990993 (MLH1) are located in the binding region of the ZNF protein family transcription factor (TF). Thus, the DNA methylation profile in the liver in HCV-induced HCC is unique and differs depending on the degree of surrounding tissue lesion – liver fibrosis or liver cirrhosis.

## Abstract (ru)

С использованием данных GSE73003 и GSE37988, представленных в базе данных GEODataSets (https://www.ncbi.nlm.nih.gov/), проведен сравнительный анализ уровня метилирования 27 578 CpG-сайтов между парными образцами опухолевой и окружающей опухоль тканями печени различной степени поражения (фиброз, цирроз) у больных HCV-индуцированной гепатоцеллюлярной карциномой (ГЦК), а также между опухолевой и нормальной тканью у больного ГЦК невирусной этиологии. Выявлено значительно меньшее число дифференциально метилированных сайтов между нормальной тканью печени и ГЦК невирусной этиологии, а также между ГЦК и фиброзом (32 и 40), чем между ГЦК и циррозом (2450 и 2304 соответственно по данным GSE73003 и GSE37988). По мере прогрессирования патологического изменения окружающей опухоль ткани уменьшается соотношение количества гипер-/гипометилированных дифференциально метилированных сай- тов в опухоли. Так, в опухолевой ткани по сравнению с нормальной/фиброзом/циррозом печени гиперметили- рованы 75/62.5/47.7 % (GSE73003) и 16 % (GSE37988) CpG-сайтов соответственно. Стойкое гиперметилирование генов ZNF154 и ZNF540, а также гипометилирование CCL20 зарегистрировано в опухолевой ткани относительно как фиброза, так и цирроза печени. Белковые продукты генов EDG4, CCL20, GPR109A и GRM8, CpG-сайты которых характеризуются изменением уровня метилирования ДНК в опухоли на фоне цирроза и фиброза, принадлежат к категории «передачи сигналов рецепторов, связанных с G-белком». Однако изменение уровня метилирова- ния «драйверных» для онкопатологии генов (АРС, CDKN2B, GSTP1, ELF4, TERT, WT1) регистрируется в опухолевой ткани на фоне цирроза печени, но не фиброза. Среди гиперметилированных в опухолевой ткани генов на фоне цирроза печени наиболее представленными биологическими путями являются процессы развития, передачи межклеточных сигналов, регуляции транскрипции, связывания с белками Wnt-пути. Гены, гипометилирован- ные в опухолевой ткани печени на фоне ее цирротического поражения, относятся к передаче обонятельных сигналов, нейроактивному взаимодействию лиганда с рецептором, кератинизации, иммунному ответу, инги- бированию сериновых протеаз и метаболизму цинка. Гиперметилированные в опухоли гены локализуются в локусе 7р15.2 в регионе кластера HOXA, а гипометилированные CpG-сайты занимают протяженные области генома в кластерах генов обонятельных рецепторов (11p15.4), кератина и кератин-ассоциированных белков (12q13.13, 17q21.2 и 21q22.11), комплекса эпидермальной дифференцировки (1q21.3), а также функционирова- ния иммунной системы – локусы 9p21.3 (кластер IFNA, IFNB1, IFNW1) и 19q13.41–19q13.42 (кластеры KLK, SIGLEC, LILR, KIR). Среди генов фиброгенеза или репарации ДНК cg14143055 (ADAMDEC1) локализован в регионе связы- вания транскрипционных факторов семейства HOX, а cg05921699 (CD79A), cg06196379 (TREM1) и cg10990993 (MLH1) расположены в области связывания транскрипционных факторов семейства белков ZNF. Таким обра- зом, профиль метилирования ДНК в печени при HCV-индуцированной ГЦК является уникальным и различается в зависимости от степени поражения окружающей ткани – фиброз или цирроз.

## Introduction

Malignant neoplasms of the liver are characterized by an increasing incidence rate worldwide (Philips et al., 2021). The highest morbidity and mortality rates are observed in East Asia and Africa, where the leading cause of hepatocellular carcinoma (HCC) is chronic viral hepatitis B and non-alcoholic fatty liver disease (NAFLD). However, in developed countries one of the main causes of HCC development is considered to be chronic viral hepatitis C (chronic HCV, CHCV); and its prevalence is high in Europe and maximal in Eastern European countries, including Russia (Goossens, Hoshida, 2015; Petruzziello et al., 2016).

The molecular mechanisms of HCC development differ significantly depending on the etiology of the disease. Thus, the hepatitis B virus (HBV) can integrate into the genome of the host hepatocyte, which leads to the direct triggering of carcinogenesis through the activation of protooncogenes and/or suppression of the activity of tumor suppressor genes (Levrero, Zucman-Rossi, 2016). In turn, the hepatitis C virus (HCV), which is an RNA virus, has limited ability to integrate into the genome of the host liver cell and realizes its carcinogenic potential by switching on a multi-stage process that leads through chronic liver inflammation and fibrosis progression to the formation and development of tumor clones. The risk of developing HCC in chronic HCV infection is directly related to the severity of liver fibrosis; it is a rare event in the initial stages of fibrosis and occurs significantly more often in patients with cirrhosis (Khatun et al., 2021).

Among the various factors determining susceptibility to HCV infection and the progression of fibrosis to HCC, the genetic and epigenetic component plays an important role. In particular, genome-wide association studies (GWAS) have identified approximately 140 loci, of which 84 are attributed to known genes, the protein products of which are involved in the response to HCV infection, antiviral therapy, spontaneous viral clearance, and the development of complications to interferon therapy (Kanz et al., 2005).

Genes, including EXO1, VCAN, KIT and MIR200C, which are associated with the development of HCV-induced HCC and considered as potential targets for pharmacotherapy, have been identified (Goossens, Hoshida, 2015; Schulze et al., 2015; Chen et al., 2021). In addition, microRNAs determined in liver tissue or serum have been shown to have prognostic value in the development of HCV-induced HCC (Aly et al., 2020; Yan et al., 2021).

There are few experimental studies of liver tissue methylome aberrations in liver pathology depending on etiological causes (Neumann et al., 2012; Hlady et al., 2014). The main data regarding viral etiology are the data obtained by comparative analysis of paired tumor and non-tumorous liver tissues in Asian patients with HCC on the Illumina Infinium Human Methylation BeadChip 27k platform (Shen et al., 2012; Mah et al., 2014; Yamada et al., 2016). A number of studies involve reanalysis of the available DNA methylation findings using additional data, including those obtained on the Illumina Human Methylation 450 BeadChip microarray from The Cancer Genome Atlas (Fan et al., 2018; Meng et al., 2018; Wang Y. et al., 2019; Jiang et al., 2020; Zhao et al., 2021).

A comparison of the lists of differentially methylated CpG sites between the analyzed liver tissues in HCC patients in different studies (Shen et al., 2012; Mah et al., 2014; Yamada et al., 2016) reveals significant similarities. For example, the list of hypermethylated genes in tumor tissue presented in the paper of (Yamada et al., 2016) overlaps by 93 % with the data of another group (Mah et al., 2014). A different picture is observed when comparing the results of reanalysis. Thus, common genes are rarely found in the lists of genes significant for the HCC development presented in various studies (Fan et al., 2018; Meng et al., 2018; Wang Y. et al., 2019; Jiang et al., 2020). This can be explained by the different criteria chosen for the reanalysis of the primary data provided in the GEO repository (Edgar et al., 2002; Barrett et al., 2013). At the same time, none of the mentioned studies took into account the etiology of HCC, and the analyzed group included both carriers of HBV or HCV and patients without viruses or their combinations.

The contribution of DNA methylation to the development of HCV- and HBV-induced HCC has been reviewed in metaanalyses including studies of targeted methylation of genes associated with liver diseases (Zhang et al., 2019, 2022). The genes hypermethylated in liver tumor tissues in HCC of various viral etiologies have been identified. However, these genes are largely common, which does not provide a complete picture of the patterns of the DNA methylation profile in the influence of hepatitis B and C viruses.

Our research team has been working on the genetic aspects of СHCV. As a result, we established the associations of polymorphisms in fibrogenesis genes and DNA repair genes with pathology and pathogenetically significant features, including stages of liver fibrosis (Goncharova et al., 2020). It is possible that there are features of the DNA methylation profile in liver tissue in the setting of fibrosis and cirrhosis induced by HCV and causing HCC.

Thus, the aim of this study was to identify changes in the DNA methylation profile, including the regions of genes involved in fibrogenesis or DNA repair, in liver tissue during the progression of HCV infection from liver fibrosis to HCC using re-analysis of primary data stored in the GEO repository

## Materials and methods

Data from several studies analyzing the profile of DNA methylation in the liver of Asian patients with HCC caused by viral hepatitis B and C on the Illumina Infinium Human Methylation BeadChip 27k platform are available in the GEO database (Table 1). For Caucasians, there is no data available on DNA methylation in HCC in the GEO repository.

**Table 1.**
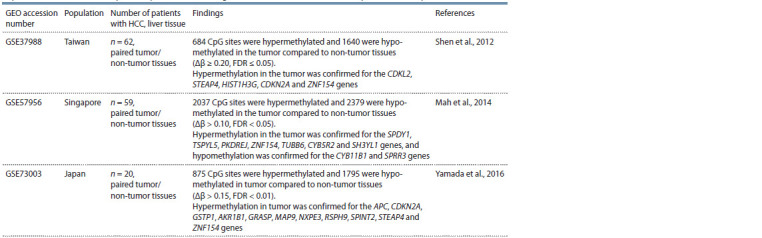
General characterization of studies related to the analysis of the DNA methylation profile in the liver in patients with HCC caused by viral hepatitis B and C using the Illumina Infinium Human Methylation BeadChip 27k

From the GSE73003 and GSE37988 datasets, we selected for analysis the patients diagnosed with СHCV by the presence of a hepatitis C virus total antibody (HCVab+) and the absence of a viral hepatitis B surface antigen (HBsAg–). From the GSE73003 dataset, we chose patients with HCV-induced HCC, in which non-tumor liver tissue was characterized by various stages of fibrotic lesion: liver fibrosis in the setting of СHCV (n = 3) and liver cirrhosis (n = 8). In addition, the study included one patient with HCC of unknown etiology, who was HCVab and HBsAg negative, in which the surrounding liver tissue was defined as normal (HCC_normal tissue/ normal tissue).

From the GSE37988 array, patients with HCV-induced HCC, in which non-tumor liver tissue was at the stage of cirrhosis (n = 6), were included in the analysis. In the present work, we did not differentiate the tissues and did not use histological sections, but relied only on the data presented in GSE37988 and GSE73003 GEODataSets (https://www.ncbi. nlm.nih.gov/).

As the GSE57956 dataset does not provide information on the etiology of the pathology, in particular hepatitis B and C viral infection, the tissue samples were not included in the present study.

In addition to the 27,578 CpG sites presented on the Illumina Infinium Human Methylation BeadChip 27k methylation array, the methylation status of fibrogenesis genes and DNA repair genes was analyzed separately. We chose genes associated with CHCV, liver fibrosis stages, the rate of fibrosis progression to liver cirrhosis and comorbid pathologies of CHCV, according to our previous studies (Goncharova et al., 2020).

Statistical data analysis was performed using lumi, limma packages in the R software environment (Bioconductor). The correction for multiple comparisons was performed using the Benjamini–Hochberg (FDR) method.

The methylation index β, which represents the ratio of the intensity of fluorescence signals of methylated alleles to the sum of fluorescence signals of methylated and unmethylated alleles, was used as a parameter of DNA methylation level. The methylation index β varies from 0 (unmethylated state) to 1 (complete methylation of all CpG sites at a given position). CpG site was considered as differentially methylated if it had a difference in the average methylation level between the groups of samples with FDR <0.05 and |Δβ| ≥ 0.2, which exceeds the microarray measurement error and complements the statistical significance of the differences by a biologically valid criterion.

Functional annotation of protein products of genes containing differentially methylated CpG sites (DMS) was performed using Web-based GEne SeT AnaLysis Toolkit programs with Weighted set cover (Liao et al., 2019) and Metascape (Zhou et al., 2019) category reductions. The categories of the genes described in terms of biological processes and molecular functions correspond to the Gene Ontology (GO) database classifier, in terms of signaling and metabolic pathways correspond to KEGG and Reactome, in terms of drug targets correspond to DrugBank, and in terms of chromosomal localization correspond to Chromosomal Location.

Additionally, we performed the genomic annotation of DMSs in fibrogenesis genes and DNA repair genes in the hepatocellular carcinoma cell line HepG2 using the UCSC Genome Browser (Kent et al., 2002). This annotation allowed us to characterize CpG sites that localize in gene promoters, open chromatin regions accessible to RNA polymerase II or transcription factor (TF) binding sites, and thereby possibly affect changes in gene expression.

## Results and discussion

Identification of DMSs and their genes between tumor and non-tumor liver tissues (normal without hepatitis C and B viruses, fibrosis and cirrhosis in the setting of CHCV) in patients with HCC

**Fig. 1.**
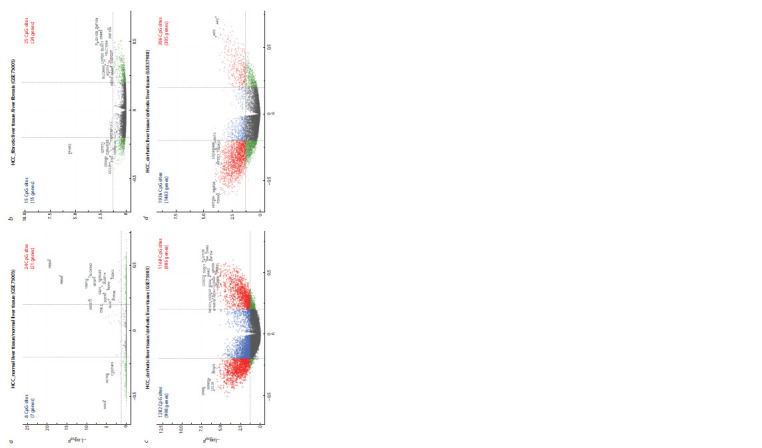
Differentially methylated CpG sites (genes) with differences in the methylation level of CpG sites |Δβ| > 0.2 и FDR < 0.05: а, between HCC with adjacent normal liver tissue/normal liver tissue without hepatitis C and B viruses (GSE73003); b, between HCC with adjacent fibrotic liver tissue and liver fibrosis in CHCV (GSE73003); c, between HCC with adjacent cirrhotic liver tissue/cirrhotic liver tissue in CHCV (GSE73003); d, between HCC with adjacent cirrhotic liver tissue/cirrhotic liver tissue in CHCV (GSE37988).

A comparative analysis of the methylation level of 27,578 CpG sites between paired samples characterized as HCC surrounded by normal tissue and normal liver tissue in a patient without hepatitis C and B viruses (GSE73003) revealed 32 DMSs, among which 24 CpG sites (21 genes) were hypermethylated and 8 CpG sites (7 genes) were hypomethylated in tumor versus normal tissue (Fig. 1, а). Two CpG sites were identified in the RBM4, SOX9 and SPAG8 genes (hypermethylated in tumor tissue), as well as in ACTA2 (hypomethylated in tumor tissue).

Twenty CpG sites with the greatest differences in methylation levels between tumor and normal liver tissues are presented in Suppl. Material 11. Most of them are located in the region of CpG islands (16 sites or 80 %). Among them are the CpG sites in the RBM4, TRIP12, BFSP1, FBP1, SGCE and PTPN4 genes, which have previously been associated with the development of HCC (see Suppl. Material 1).


Supplementary Materials are available in the online version of the paper: http://vavilov.elpub.ru/jour/manager/files/Suppl_Goncharova_Engl_27_1.pdf


Forty differentially methylated sites were identified in HCC surrounded by fibrotic tissue versus fibrosis in CHCV (GSE73003) (see Fig. 1, b). In the liver tumor tissue, 25 CpG sites (24 genes) were hypermethylated compared to the fibrotic tissue and 15 CpG sites (15 genes) were hypomethylated. Significant changes in methylation levels during oncotransfor- mation of fibrotic liver tissue were shown for the CpG sites of the ZNF154, DNM3, DLEC1, LYPD3, DDX49, NEFH, CCL20 and NNMT genes, which were previously associated with HCC development (see Suppl. Material 1). Moreover, the most significant hypermethylation in the tumor versus fibrosis was detected for two CpG sites located in the region of the CpG island in the 1st exon of the ZNF154 gene (Δβ = 0.593–0.596, FDR <0.01).

Of all the differentially methylated genes (DMGs), only the CCL20 protein product is a proangiogenic chemokine that is highly upregulated in cells infected with HCV and induces endothelial cell invasion and migration during HCC formation (Benkheil et al., 2018). The cg21643045 site in the CCL20 gene, located in exon 1, was hypomethylated in tumor tissue compared to fibrotic tissue (Δβ = –0.382, FDR = 0.0235).

A comparison of DNA methylation level between paired samples of liver tissues (tumor and cirrhosis) in CHCV (GSE73003) revealed 2450 DMSs (see Fig. 1, c). In tumoraffected liver tissue versus non-tumor tissue, 1168 CpG sites (886 genes) were hypermethylated and 1282 CpG sites (998 genes) were hypomethylated.

Of the twenty CpG sites of genes that showed the most significant changes in methylation level during oncotransformation of liver tissue affected by cirrhosis, the GRM8, DNM3, DLEC1, ZNF154, WNK2, MFAP5, FOXD3, NEFH, MTNR1B, CCL20 and RAB31 genes were associated with HCC development (see Suppl. Material 1). Moreover, cg21790626 in the ZNF154 gene and cg21643045 in the CCL20 gene were hyper- and hypomethylated, respectively, in tumor tissue versus cirrhotic tissue (Δβ = 0.598, FDR = 3.10×10–7 and Δβ = –0.459, FDR = 1.43×10–6).

A comparative analysis of the methylation level of 27,578 CpG sites between paired samples of tumor and nontumor liver tissue in the setting of HCV-induced liver cirrhosis (GSE37988) revealed 2304 DMSs (see Fig. 1, d ). In the liver tumor tissue versus cirrhotic tissue, 386 CpG sites (305 genes) were hypermethylated and 1936 CpG sites (1483 genes) were hypomethylated.

The genes and CpG sites that showed the most significant changes in the methylation level during oncotransformation of liver tissue affected by cirrhosis according to GSE37988 are presented in Suppl. Material 1. Among them, the MAGEA3, APC, AKT3, MMP26 and WFDC1 genes are associated with HCC according to previous studies (see Suppl. Material 1). In contrast to the GSE73003, a smaller proportion of CpG sites (7 out of 20, or 35 %) were located in the region of CpG islands. Moreover, only two of them, cg16970232 and cg24332422 in the APC gene, were hypermethylated in tumor tissue versus cirrhotic tissue (Δβ = 0.730, FDR = 1.0×10–4 and Δβ = 0.581, FDR = 1.2×10–4).

Characterization of common DMGs between tumor and non-tumor liver tissues (normal without hepatitis C and B viruses, fibrosis and cirrhosis in the setting of CHCV) in patients with HCC

A comparison of the lists of genes containing DMSs between tumor and non-tumor tissues in patients with HCC, depending on the degree of tumor-adjacent liver tissue damage, revealed that the ZNF154, DNM3, FLJ21159, DLEC1, CCDC37, NEFH, CCL20 and KRTAP11-1 genes are among the top ones with maximum differences in the methylation level of CpG sites between the tissues (see Suppl. Material 1).

**Fig. 2.**
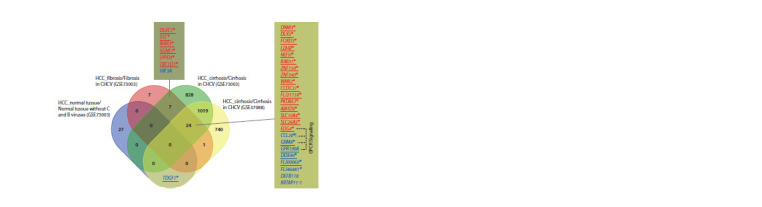
Venn diagram showing the number of total DMGs between the tumor and adjacent liver tissue of different lesion degrees (normal without C and B viruses, fibrosis and cirrhosis in CHCV) in patients with HCC. Blue/red – hypo-/hypermethylated genes in tumor tissue versus non-tumor tissue; underlined – location of DMSs in the region of CpG island; */! – gene involved in HCC/HCC in CHCV.

The differentially methylated genes between tumor tissues and liver fibrosis/cirrhosis (GSE73003) are characterized by the presence of seven common genes, six of which are hypermethylated in the tumor regardless of the degree of surrounding tissue damage (Fig. 2). Five of the seven DMSs in common genes are located within CpG islands. The DLEC1, SST, IRAK3, SGNE1, LYPD3 and TBC1D1 genes have previously been shown to be associated with the development of HCC, and the DLEC1, IRAK3 and SGNE1 genes were hypermethylated in the tumor (Qiu et al., 2008; Kuo et al., 2015; Meng et al., 2018), which is consistent with the results of the present study.

There are 24 DMGs common to tumors in the presence of fibrosis and cirrhosis from the two datasets (GSE73003 and GSE37988). Among them, 16 DMGs (66.7 %) are hypermethylated and located in the CpG island region (see Fig. 2). An association with HCC development has previously been shown for 21 genes: DNM3 was downregulated and FOXD3, LDHB, NEFH, ZNF154, FLJ21159, PKDREJ, ABHD9 and WNK2 were hypermethylated in tumor tissue (Shen et al., 2012; Revill et al., 2013; Liu Z. et al., 2016; Meng et al., 2018; Miller et al., 2021). The CCDC37, CCL20, DNM3, ZNF154 and ZNF540 genes overlap with the list of twenty DMGs in HCC regardless of etiology (Shen et al., 2012).

Among the eight genes hypomethylated in the tumor, for CCL20, DDX49 and GRM8, the increased expression in blood serum and/or tumor tissue in patients with HCC was previously demonstrated, including upregulation of CCL20 in HCC in the setting of CHCV (Benkheil et al., 2018; Dai et al., 2021; Gao et al., 2022)

We performed a functional annotation of 24 common DMGs between the tumor and the adjacent liver tissue of various degrees of damage using the Metascape resource (see Fig. 2). It showed the association of hypermethylated (EDG4) and hypomethylated genes (CCL20, GPR109A and GRM8) with processes of signaling by G-protein-coupled receptors (R-HSA-372790). Moreover, the expression of the GRM8 gene in tumor tissue negatively correlates with the survival of patients with HCC, and its methylation level is included in the panel of genes important for disease prediction (Gao et al., 2022). It is thought that GPCRs play the role of oncomodulators, the aberrant expression of which alters various normal signaling pathways in the cells, disrupting angiogenesis, invasion, migration, metastasis, and immune response in HCC initiation and progression, which makes them attractive molecular therapeutic targets (Peng et al., 2018).

The present study revealed hypermethylation of the CpG sites of the ZNF154 and ZNF540 genes encoding zinc finger proteins in liver tumor tissue compared to fibrosis and cirrhosis (see Fig. 2). Some proteins of this category are included in the signature of prognostic markers of survival of patients with HBV-induced HCC and are the top hypermethylated genes in HCC of various etiologies (Shen et al., 2012; Wang X. et al., 2021). An analysis of the expression of these genes in the liver showed that in HCC of various etiologies, transcription repression of many zinc finger proteins ZNF is observed (Gonçalves et al., 2022). It is likely that in HCV-induced HCC, the zinc finger protein genes, in particular ZNF154 and ZNF540, can be promising early markers of oncotransformation, beginning with fibrosis, and not only in the setting of liver cirrhosis.

None of the genes from the list of 24 common DMGs between tumor and adjacent liver tissues in fibrosis and cirrhosis in the setting of CHCV from the two data sets (GSE73003 and GSE37988) were included in the list of known molecular “drivers” of malignancies, including HCC (Hlady et al., 2014; Bailey et al., 2018; Cai et al., 2020; Molina-Sánchez et al., 2020; Zhang et al., 2022). However, such genes are found among DMGs between tumor and cirrhotic tissues. In particular, CpG sites within the CpG islands of the promoters of the АРС, CDKN2B, GSTP1, ELF4 и and TERT genes were hypermethylated in tumor tissue, and various CpG sites of the WT1 gene were characterized by multidirectional changes in their methylation levels.

Functional annotation of DMGs between tumor and non-tumor liver tissues (normal without hepatitis C and B viruses, fibrosis and cirrhosis in the setting of CHCV) in patients with HCC

In terms of the most represented biological pathways and basic molecular functions, the genes harboring hypo- and hypermethylated CpG sites in tumor tissue, compared to cirrhotic tissue in patients with HCC in the setting of CHCV, are similar between the GSE73003 and GSE37988 datasets (Suppl. Material 2). Thus, for genes the CpG sites of which are hypermethylated in tumor tissue, biological processes related to development (GO:0007399, GO:0009790, GO:0048468, FDR < 2.2×10–16) and cell-cell signaling (GO:0007267, FDR < 2.2×10–16, see Suppl. Material 2) are most represented. These results are partially consistent with (Shen et al., 2012) data, where developmental processes are distinguished among the most significant in HCC of various etiologies. Genes containing hypermethylated CpG sites in HCV-induced HCC are similar in molecular functions to genes identified in HCC of various etiologies (Shen et al., 2012) and include transcription regulation and DNA binding (GO:0003700; GO:0140110, GO:0003677, FDR < 0.0002), as well as Wnt-protein binding (GO:0017147, FDR = 1.3×10–4).

The hypermethylated genes are located on chromosome 7 (7р15.2) in the region of the HOXA cluster (FDR = 2.3×10–5, see Suppl. Material 2). Previously, identification of DNA methylation signature in liver tissue in HCC showed that 39 out of 214 CpG sites were associated with altered gene expression. This includes genes located in the сhr7:27144326– 27145664 region in close proximity to homeobox transcription factors (HOXA6, HOXA3, HOXA5, HOXA7 and HOXA4) that are involved in oncogenesis, cell proliferation and migration (Gonçalves et al., 2022).

Hypomethylated genes in HCV-induced HCC are mainly related to the following biological processes: immune and defense responses (GO:0006955, GO:0006952, FDR <2.2×10–16); G protein-coupled receptor signaling pathway (GO:0007186, FDR < 6.0×10–10); epithelial cell differentiation (GO:0030855, FDR <2.2×10–16, see Suppl. Material 2), which is partially consistent with the data obtained for HCC of various etiologies (Shen et al., 2012).

According to the molecular functions of hypomethylated genes in HCV-induced HCC of various etiologies (Shen et al., 2012), on the one hand, similarities are revealed with respect to several categories, such as binding to receptors of various antigens, and on the other hand, the activity of peptidase inhibitors, including serine-type peptidase, is noted only in HCV-induced carcinoma (see Suppl. Material 2). The serine protease inhibitor secreted by liver tumor cells (SPINK1 or LC-SPIK) is now known to be a protein that significantly increases in the blood serum of individuals with HCC of viral etiology (Lu et al., 2020).

According to the KEGG and Reactome databases, the most significant molecular pathways for genes hypomethylated in the tumor were olfactory transduction (hsa04740, FDR < < 2.2×10– 16), cytokine-cytokine receptor interaction (hsa04060, FDR < 7.8×10–7) and neuroactive ligand-receptor interaction (hsa04080, FDR < 0.0007); signaling by G protein-coupled receptors (GPCRs) (R-HSA-372790, FDR < 3.5×10– 6), keratinization (R-HSA-6805567, FDR < 2.2×10– 16) and immune system (R-HSA-168256, FDR < 3.4×10–6, see Suppl. Ma-terial 2). Apparently, this is due to the fact that DNA hypomethylation in the tumor spreads over extended genome regions in the gene clusters of olfactory receptors (11p15.4), keratin and keratin-associated proteins (12q13.13, 17q21.2 and 21q22.11), epidermal differentiation complex (1q21.3), as well as immune system functioning – loci 9p21.3 (IFNA, IFNB1, IFNW1 cluster) and 19q13.41–19q13.42 (LILR, KIR, KLK, ZNF, SIGLEC clusters, see Suppl. Material 2).

Disruption of epigenetic regulation of the immune system is a common feature in cancers of various localizations (Berglund et al., 2021). Olfactory transduction and neuroactive ligand-receptor interaction are part of the G protein-coupled receptor signaling pathway, the enrichment of which is also common in malignant neoplasms (Wei et al., 2012). Ectopic expression of olfactory receptor genes, associated with epigenetic mechanisms among others, seems to provide invasiveness and metastasis of tumor cells in the late stages of malignancy (Fessahaye et al., 2021). Disruption of the keratinization process is a less frequently reported event in tumor. For it, an association with the DNA hydroxymethylation level in head and neck cancer depending on the carriage of the human papillomavirus is shown (Liu S. et al., 2020), as well as the enrichment of hypomethylated genes in breast cancer (Holm et al., 2016).

The DrugBank database indicates that hypomethylated genes in HCV-induced HCC are involved in zinc metabolism (DB01593); and zinc supplementation may be recommended to reduce the risk of HCC after HCV eradication with directacting antiviral agents (Hosui et al., 2021). It is possible that there is an association between zinc deficiency and hypomethylation of DNA in individual genes (Azimi et al., 2022).

The profile of methylation of fibrogenesis genes and DNA repair genes

Ten differentially methylated CpG sites were identified among the genes the protein products of which are involved in the processes of fibrogenesis or DNA repair from the category of genes previously shown to be associated with liver diseases in the studies of our research group (Table 2). The cg03876618 site of the IGFBP7 gene and the cg14323109 site of the KDR gene located in the CpG island regions were hypermethylated in the tumor compared to the surrounding cirrhotic tissue. The CpG sites of the ADAMDEC1, CD79A, MMP3 and TREM1 genes were differentially methylated according to the GSE37988 and GSE73003 datasets (see Table 2).

**Table 2.**
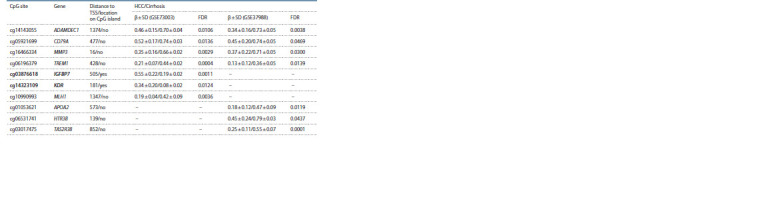
DMSs of genes involved in fibrogenesis and DNA repair between tumor and cirrhotic liver tissue in patients with HCC

Genomic annotation using the UCSC Genome Browser showed that in the hepatocellular carcinoma cell line HepG2, the active promoter contains cg01053621 (APOA2) and cg10990993 (MLH1); and cg03876618 (IGFBP7), cg14323109 (KDR), cg16466334 (MMP3), cg06196379 (TREM1) и cg01053621 (APOA2) are localized in the RNA polymerase II subunit A binding regions

The cg14143055 site of the ADAMDEC1 gene, hypomethylated in tumor tissue, is localized in the binding region of HOX family transcription factors, which play an important role in oncogenesis of various tumors, including HCC (Gonçalves et al., 2022). At the same time, HCV infection and virus core protein expression trigger HOX gene activation (Kasai et al., 2021), which may be one of the factors in the development of HCV-induced HCC

CpG sites hypomethylated in tumor tissue in HCV-induced HCC – cg05921699 (CD79A), cg06196379 (TREM1), cg10990993 (MLH1) – are located in the binding region of the TFs, representing the zinc finger protein (ZNF) family. ZNF, in addition to regulation of transcription, induce protein-protein interactions, post-transcriptional regulation, lipid metabolism, immune responses, and affect the development of many forms of cancer, including HCC (Li et al., 2022).

In conclusion, it should be noted that our study has a limitation due to the small size of samples of patients with normal and fibrotic tissues surrounding the tumor in CHCV, since in most cases HCC develops in the setting of cirrhotic tissue and other cases are observed much less frequently. The study did not consider the intratumoral and cellular heterogeneity of tissues, which is closely related to the DNA methylation profile (Hlady et al., 2017). Taking into account the fact that the focus of the study was to analyze the DNA methylation profile in the liver in HCV-induced HCC, it is difficult to unambiguously identify CpG sites specific to this pathology. A methodological limitation is the impossibility of distinguishing between DNA methylation and DNA hydroxymethylation, since it undergoes bisulfite modification before hybridization on a methylation profiling microarray.

## Conclusion

A comparative analysis of the DNA methylation profile in the liver of patients with HCC between tumor and non-tumor tissues with various degrees of lesion (normal tissue, HCVinduced fibrosis, HCV-induced cirrhosis) showed a significantly lower number of DMSs between HCC and normal tissue without hepatitis C and B viruses/liver fibrosis in CHCV (32 and 40) than between HCC and liver cirrhosis in the setting of HCV in the GSE73003 and GSE37988 datasets (2450 and 2304, respectively).

Based on the fact that the severity of fibrosis correlates with liver function and cirrhosis is the main risk factor for HCC development (Roehlen et al., 2020), we can expect normal and fibrotic liver tissue to be maximally distant from HCC by their epigenetic profile and, as fibrosis progresses to cirrhosis, the number of DMSs between the tumor and the surrounding tissues will decrease. Nevertheless, we see the opposite pattern: the more severe the lesion of the liver tissue surrounding the tumor, the greater the differences in DNA methylation levels observed between them. It is possible that normal liver tissue or tissue with minimal fibrotic lesion helps to restrain the functional imbalance of tumor genome, causing minimal differences in the DNA methylation profile between these tissues. This assumption is indirectly confirmed by the fact that changes in the methylation level of the “driver” genes for HCC are registered in the setting of cirrhosis, but not fibrosis.

As the pathological changes in the liver tissue surrounding the tumor progress, the ratio of hyper-/hypomethylated DMSs in the tumor decreases. Thus, in patients with HCC, 24 CpG sites, or 75 % of all DMSs, are hypermethylated in tumor tissue compared to normal tissue. Compared to liver tissue affected by fibrosis in the setting of CHCV, 25 out of 40 DMSs, or 62.5 %, are hypermethylated in tumor tissue. When the liver tissue surrounding the tumor is cirrhotic, the number of hypermethylated CpG sites in tumor tissue versus the comparison group is 47.7 and 16 % (GSE73003 and GSE37988, respectively). Previous studies have also revealed the predominance of hypomethylated CpG sites in extended genome regions, including those in the region of genes and intergenic regions, in HCC tumor tissue versus the surrounding cirrhotic liver tissue (Shen et al., 2012; Hlady et al., 2014; Yamada et al., 2016; Yan et al., 2021). The present study shows for the first time that in patients with HCC the tumor in the setting of unaffected liver tissue and with liver fibrosis in CHCV is characterized by a greater proportion of hypermethylated CpG sites, while the number of hypomethylated sites increases in tumor tissue in cirrhosis.

The studies of the profile of gene methylation in the liver in HCC focus on hypermethylated genes, including genes of the ZNF and HOX families, among which the search for markers significant for disease development is performed. At the same time, a comparative analysis showed that in HCV-induced HCC, a greater number of hypermethylated CpG sites were observed in tumor tissue only compared to the surrounding tissue with features of fibrosis. In the case when the tissue surrounding the tumor represents liver cirrhosis, most of the loci in the tumor tissue are hypomethylated, which appears to be a late event that occurs during the transition from the fibrotic damage of liver tissue to malignant transformation.

In this regard, in HCV-induced HCC, attention should also be paid to hypomethylated loci, which, as shown in this study, belong to GPCR proteins (CCL20, GPR109A and GRM8), localized in the binding sites of such TFs as HOX (ADAMDEC1), ZNF (CD79A, MLH1) or in the region of serine protease inhibitor genes, one of which – SPINK1 – is currently considered as a marker capable of detecting HCC of viral etiology at an early stage. In addition, in our work, hypomethylated DMSs were localized in genes associated with zinc metabolism, which is known to play a role in the pathogenesis of many diseases, including HCC.

Thus, the functional state and lesion degree of the tissue surrounding the tumor must be taken into account in studies evaluating the DNA methylation profile in the liver in HCC, since the DMGs spectrum differs significantly between tumor/ non-tumor tissue pairs, depending on whether it is relatively normal or with features of fibrosis or cirrhosis. To identify prognostic markers of HCC, including liquid biopsies, the etiology of the disease should be considered, since the spectrum of DMSs and DMGs of HCV-induced HCC only partially overlaps with those identified in the analysis of this pathology of other nature.

## Conflict of interest

The authors declare no conflict of interest.
